# Lifestyle, psychological stress, and incidence of adolescent constipation: results from the Toyama birth cohort study

**DOI:** 10.1186/s12889-020-10044-5

**Published:** 2021-01-06

**Authors:** Masaaki Yamada, Michikazu Sekine, Takashi Tatsuse, Yuko Fujimura

**Affiliations:** 1 Department of Epidemiology and Health Policy, School of Medicine, University of Toyama, Toyama, Japan. 2630 Sugitani, Toyama, 930-0194 Japan; 2 Department of Community Medicine and Health Promotion, University of Toyama, Toyama, Japan. 2630 Sugitani, Toyama, 930-0194 Japan

**Keywords:** Lifestyle change, Bowel movement, Physical activity, Breakfast, Predisposing factors

## Abstract

**Background:**

We aimed to clarify the predisposing factors for adolescent constipation in a longitudinal study, because while factors associated with childhood constipation have been reported, prospective studies on the incidence of constipation are lacking.

**Methods:**

We enrolled 5540 adolescents aged 12 to 13 years from the Toyama Birth Cohort Study—a community-based prospective study examining children’s health. The incidence of constipation, defined as bowel movement frequency of less than once every 2 days, was surveyed during the three-year period from baseline (grade 4) to follow-up (grade 7). Multivariate logistic regression analyses were performed to explore the association between the incidence of adolescent constipation and their lifestyle variables.

**Results:**

A total of 261 adolescents (4.7%) developed constipation during the three-year period. Female sex (odds ratio [OR] = 2.62,) overweight (OR = 0.60), and infrequent intake of fruits (OR = 1.50) at baseline were associated with the incidence of constipation. Furthermore, factors related to lifestyle changes and psychological status such as skipping breakfast (OR = 1.73), becoming physically inactive (OR = 1.55), and being persistently irritated (OR = 1.80) were significantly associated with the incidence of constipation.

**Conclusion:**

Our prospective study demonstrated that female sex, insufficient fruit intake, and deteriorating lifestyles such as skipping breakfast and becoming inactive during the 3-year period were associated with the incidence of adolescent constipation. Beyond anecdotal, maintaining a healthy lifestyle is recommended to reduce the incidence of adolescent constipation.

**Supplementary Information:**

The online version contains supplementary material available at 10.1186/s12889-020-10044-5.

## Background

Constipation is common, with a prevalence ranging from 0.5 to 32.2% in children aged 0 to 18 years [1]. In many cases, the prognosis is not always good [2, 3]. For instance, Loening-Baucke reported that among 94% of patients, constipation recurred immediately after laxative discontinuation [2]. Bongers et al. showed that constipation persisted into adulthood in approximately 25% of children [3]. Moreover, constipation has a negative impact on both individuals and society as a whole. It causes acute abdominal pain and decreases patients’ quality of life [4, 5]. Additionally, it results in 2.5-million physician visits annually and contributes to considerable healthcare financial burden in the American society [6]. Thus, childhood constipation should be considered a major public health problem.

Several risk factors, such as insufficient fiber intake, genetic predisposition, physical inactivity, and psychological stress, have been identified for childhood constipation [1, 7], but most were inferred from cross-sectional studies. We previously reported that psychological stress and less frequent parental interaction were as strongly associated with constipation as dietary factors [8]. To clarify the predisposing factors, a prospective cohort study is needed to assess the incidence of constipation among children, especially adolescents who are starting to establish their lifestyle, which they will continue during into adulthood.

The existing few prospective studies on childhood constipation have primarily focused on its prognoses [2, 3, 9], such as recovery or success rate in clinical settings. Bongers et al. demonstrated that the onset of childhood constipation at an older age was associated with poor clinical outcomes in adulthood [3]. However, prospective studies exploring constipation incidence among older children are lacking. We hypothesized that elucidating predisposing factors of adolescent constipation could decrease the incidence of constipation, thereby easing the social burden placed on the health care system.

Thus, we performed a school-based prospective study among Japanese adolescents to provide data on the incidence of adolescent constipation. To our knowledge, this is the first and largest study assessing the predisposing factors of adolescent constipation.

## Methods

### Study participants

This study included children from the Toyama Birth Cohort Study, a longitudinal survey (a repeated open cohort style) examining lifestyle and health in 10,438 children born in Toyama Prefecture, Japan, between April 2, 1989 and April 1, 1990. The school children had been examined via a questionnaire every 3 years from Phase 1 in 1992 to Phase 5 in 2005. The details of the study have been described elsewhere [8, 10–13]. The institutional review board at Toyama Medical and Pharmaceutical University (current University of Toyama) and the prefecture education authorities approved the current study.

### Questionnaire at baseline

The Toyama Birth Cohort Study Phase 3 was conducted in July 1999, when the cohort of children were in grade 4 in elementary schools (aged 9–10 years) [8, 10]. We defined Phase 3 as the baseline survey. The questionnaire included four main areas: lifestyle, including physical activity and food consumption frequency; psychological status; child-parent interaction; and health status such as anthropometric data and bowel movements. (Additional file 1) Children answered questions on their lifestyle, psychological status, child-parent interaction at home, and bowel movements, meanwhile, their parents responded regarding their children’s food consumption frequency and anthropometric data. The lifestyle items included breakfast (the answer was dichotomized into “every day” or “skipping”), physical activity (“active” or “inactive”), and watching TV on weekdays (< 2, < 3, or ≥ 3 h). We divided sleep duration into two categories (< 8 or ≥ 8 h), referring to the average sleep duration of Japanese elementary school children (8.5 h per night) [14]. We defined < 8 h sleep a day in grade 4 children as “short sleep.” We also asked food consumption frequency about fruits and vegetables. Our lifestyle questionnaire was validated in previous studies. The frequent physical activity significantly correlated with increased energy expenditure measured by the Actiwatch-L (Mini Mitter Company, Inc., Bend, OR) [15]. The correlation coefficient between subjective and objective records of sleep duration was 0.97 (*p* < 0.001) [16] . Weight and height were measured by trained school nurses at the children’s schools and body mass index (BMI) was calculated. Age- and sex-specific cutoff points (19.46 kg/m^2^ for boys and 19.45 kg/m^2^ for girls), which were equivalent to the adult BMI value of 25 kg/m^2^, were used to define children who were overweight [17]. We asked three questions on children’s psychological status and parent-children’s interaction. The answers were from four options: often, sometimes, rarely, and none; then, the former and latter two responses were combined into a category, “not rare(ly)” and “rare(ly)” in Table 3, respectively.

### Definition of constipation

The definition of constipation ranges from self-reported to the fulfillment of clinical criteria including bowel movement frequency and symptoms [6, 7]. In our epidemiological study, we focused on only bowel movement frequency. The answer was classified into three categories: at least once daily, once every 2 days, or less frequently than once every 2 days. Then, bowel movements less frequently than once every 2 days were defined as constipation, because this corresponds to the Rome IV criterion of “two or fewer defecations in the toilet per week” [7, 18].

### Questionnaire at follow-up

In July 2002, three years after the baseline survey, we followed up the children at the Toyama Birth Cohort Study Phase 4, when they were adolescents in grade 7 (aged 12 to 13 years) [11]. Lifestyle at follow-up included skipping breakfast, physical activity, watching TV, and sleep duration. Sleep duration was dichotomized into two categories (< 7 or ≥ 7 h) on weekdays, and < 7 h sleep duration was defined as “short sleep” considering children’s growth. Adolescents with BMI values exceeding the cutoff points (21.56 kg/m^2^ for male and 22.14 kg/m^2^ for female) were classified as overweight [17]. Regarding psychological status and parent-children interaction, the same three questions at baseline were used.

### Changes in lifestyle, psychological status, overweight, and parent-children interaction

To clarify the importance of changes in lifestyle, psychological status, overweight, and child-parent interaction during the three years on the incidence of adolescent constipation, we divided the changes into four categories, such as “healthy to healthy,” “healthy to unhealthy,” “unhealthy to healthy,” or “unhealthy to unhealthy.”

### Statistical analysis

Baseline characteristics of the distributions of lifestyle, overweight, psychological status, and children-parent interaction among the cohort of children were reported. Comparisons between adolescents with complete follow-up data and those who dropped out were conducted by chi-squared tests. Next, we conducted logistic regression analysis to clarify the predisposing factors of constipation and to assess the impact of changes in lifestyle, psychological status, overweight, and children-parent interaction on constipation incidence. We calculated crude and adjusted odds ratios (ORs) and 95% confidence intervals (CIs). We adopted a forced-entry method and all independent variables (shown in Tables 2 and 3) were simultaneously included in the multivariate models. All analyses were performed with SPSS, version 25.0 J (SPSS, Chicago, IL, USA). A two-tailed *p*-value < 0.05 was considered to be significant.

## Results

**Fig. 1:**
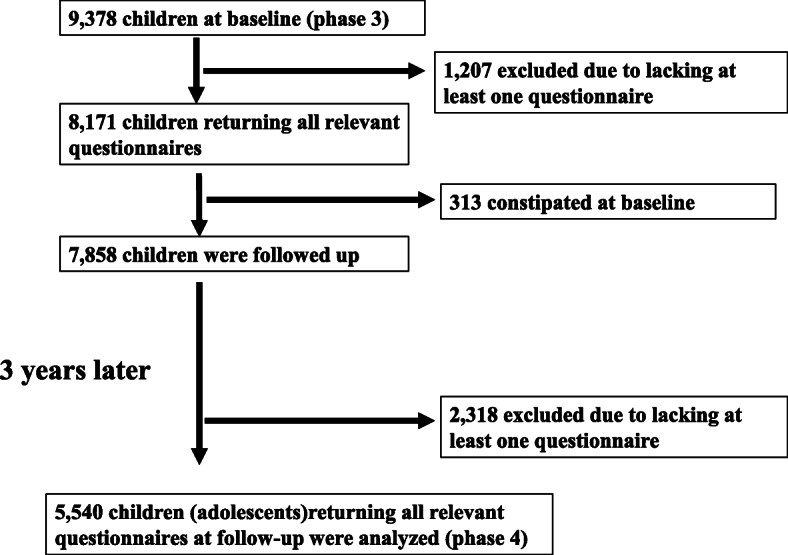
The flow of participants on the cohort study

In total, 9378 cohort children were included in the baseline survey. After excluding children who did not return all relevant questionnaires and had already been constipated, 7858 children were followed. The flow of participants in the study is shown in Fig. 1. Finally, 5540 children (male 2844, 51.3%) who answered all questionnaires at the follow-up survey were deemed eligible (follow-up rate, 70.5%). Characteristics of children at baseline and (adolescents) follow-up are presented in Table 1. Follow-up bias between adolescents who were finally included in our analyses and those excluded was assessed on the incidence of constipation and lifestyle factors. There were no significant differences in the incidence of constipation, consumption of vegetables and fruits, and physical activity between the two groups.

**Table 1:** Characteristics of children at baseline (Phase 3) and follow-up (Phase 4)

	Mean age (SD)	Baseline 9.7 (0.29)	Follow-up 12.7 (0.28)	Chi-square test
	***n*** = 5540	%	%	p
Female (girl)		48.7		
Skipping breakfast		6.3	11.7	< 0.001
Sleep duration	< 7	0.3	17.9	< 0.001
(hour)	< 8	5.4	45.3	
	< 9	46.4	29.7	
	9 or more	47.9	7.2	
Exercise	inactive	23.1	31.9	< 0.001
TV viewing	< 2	63.5	52.3	< 0.001
(hour)	< 3	25.4	27.2	
	3 or more	11.0	20.5	
Overweight	yes	15.1	15.4	0.673
Fruits consumption, daily		37.3	–	n.a
	3–5 times/week	36.4	–	
	0–2 times/week	26.3	–	
Vegetable consumption, daily		63.1	–	n.a
	3–5 times/week	25.4	–	
	0–2 times/week	11.5	–	
Irritability	rare	31.7	25.7	< 0.001
	sometime	54.8	53.1	
	often	13.6	21.2	
Unwilling to attend school, rare		64.2	63.2	0.069
	sometime	29.5	27.8	
	often	6.3	9.0	
Interaction with parents, often		26.8	16.4	< 0.001
	sometime	41.4	34.8	
	rare	31.8	48.8	

*n.a* not applicable

Table 2 shows the logistic regression analysis results on overall constipation incidence. Over three years, 261 adolescents developed constipation (incidence, 4.7%). In the univariate analysis, female sex, watching TV for < 3 h, infrequent consumption of fruits, being overweight (inversely), and frequent irritability at baseline were associated with constipation incidence. In the multivariate analysis, female sex (OR 2.62; 95% CI, 1.99–2.62), being overweight (OR 0.60; 95% CI, 0.40–0.91), and infrequent consumption of fruits (OR 1.50; 95% CI, 1.08–2.09 for 0–2 times/week) remained factors significantly associated with constipation incidence. ORs for the difference in constipation between males and females were hardly changed in the univariate and multivariate analyses.

**Table 2:** Lifestyle, overweight, and psychological status at baseline and incidence of constipation

	n = 5540	constipation (%)	univariate		multivariate		
			OR	95%CI	OR	95%CI	p
**Female (/male)**		**6.8/2.7**	**2.63**	**(2.01–3.45)**	**2.62**	**(1.99–3.46)**	**< 0.001**
Skipping breakfast (/ every day)		5.2/4.7	1.11	(0.68–1.82)	1.05	(0.63–1.73)	0.863
Sleep duration	< 8	5.7	1.29	(0.78–2.16)	1.28	(0.76–2.14)	0.360
(hour)	< 9	4.9	1.10	(0.85–1.42)	1.09	(0.84–1.42)	0.515
	9 or more	4.4	1		1		
Exercise	inactive(/ active)	5.0/4.6	1.09	(0.82–1.45)	1.04	(0.77–1.4)	0.790
TV viewing	< 2	4.2	1		1		
(hour)	< 3	5.7	**1.36**	**(1.03–1.80)**	1.24	(0.93–1.64)	0.140
	3 or more	5.2	1.25	(0.84–1.85)	1.10	(0.74–1.65)	0.633
**Overweight**	**yes (/ no)**	**3.1/5.0**	**0.61**	**(0.40–0.92)**	**0.60**	**(0.40–0.91)**	**0.016**
**Fruits consumption, daily**		4.0	1		1		
	3–5 times/week	4.9	1.23	(0.92–1.66)	1.31	(0.97–1.78)	0.082
	**0–2 times/week**	**5.4**	**1.37**	**(1.00–1.87)**	**1.50**	**(1.08–2.09)**	**0.017**
Vegetable consumption, daily		4.8	1		1		
	3–5 times/week	4.2	0.87	(0.64–1.18)	0.80	(0.59–1.10)	0.166
	0–2 times/week	5.3	1.12	(0.76–1.63)	1.05	(0.71–1.56)	0.814
Irritability	rare	3.9	1		1		
	sometime	4.8	1.24	(0.92–1.66)	1.18	(0.88–1.60)	0.269
	often	6.1	**1.59**	**(1.09–2.34)**	1.41	(0.94–2.12)	0.101
Unwilling to attend school, rare		4.4			1		
	sometime	5.1	1.18	(0.90–1.55)	1.09	(0.82–1.45)	0.548
	often	6.6	1.55	(0.99–2.44)	1.47	(0.91–2.37)	0.114
interaction with parents, often		5.2	1		1		
	sometime	4.4	0.83	(0.61–1.13)	0.87	(0.64–1.19)	0.380
	rare	4.8	0.91	(0.67–1.25)	0.97	(0.70–1.35)	0.874

*OR* odds ratio, *CI* confidence interval

Variables with a significant relationship to constipation are shown in bold

Table 3 shows the impact of the changes in lifestyle, overweight, psychological status, and child-parent interaction during the three years on the incidence of adolescent constipation from the logistic regression analysis. In the univariate analysis, breakfast with “every day to skipping,” physical activity with “active to inactive” and “with inactive to inactive,” sleep duration with “not short to short,” TV viewing with “≥3 h to ≥3 h,” BMI with “overweight to overweight,” irritability with “rare to not rare” and with “not rare to not rare,” and unwillingness to attend school with “not rare to not rare” were associated with constipation incidence. In the multivariate analysis, breakfast with “every day to skipping” (OR 1.73; 95% CI, 1.20–2.49), physical activity with “active to inactive” (OR 1.55; 95% CI, 1.14–2.11), BMI with “overweight to overweight” (OR 0.39; 95%CI, 0.21–0.70), and irritability with “not rare to not rare” (OR 1.80; 95% CI, 1.03–3.13) remained factors significantly associated with constipation incidence. Frequency of interaction with parents did not have a significant impact on adolescent constipation.

**Table 3:** Impact of changes in lifestyle, overweight, and psychological status on incidence of constipation

	n = 5540	constipation	univariate		multivariate		
	Baseline to 3 years later	%	OR	95%CI	OR	95%CI	P
Breakfast	every day to everyday	4.3	1		1		
	**every day to skipping**	**8.6**	**2.09**	**(1.47–2.97)**	**1.73**	**(1.20–2.49)**	**0.003**
	skipping to every day	3.3	0.77	(0.34–1.76)	0.72	(0.31–1.67)	0.442
	skipping to skipping	7.1	1.72	(0.94–3.14)	1.51	(0.81–2.81)	0.199
Exercise	active to active	3.8	1		1		
	**active to inactive**	**7.3**	**2.01**	**(1.49–2.70)**	**1.55**	**(1.14–2.11)**	**0.006**
	inactive to active	3.5	0.93	(0.57–1.53)	0.92	(0.56–1.52)	0.743
	inactive to inactive	6.1	**1.66**	**(1.17–2.35)**	1.42	(0.98–2.04)	0.057
Sleep	not short to not short	4.3	1		1		
	not short to short	6.6	**1.57**	**(1.16–2.14)**	1.26	(0.92–1.72)	0.228
	short to not short	5.6	1.34	(0.72–2.51)	1.26	(0.67–2.39)	0.478
	short to short	5.7	1.36	(0.63–2.97)	1.07	(0.48–2.37)	0.865
TV	< 3 to < 3	4.1	1		1		
(hour)	< 3 to ≥3	5.2	1.30	(0.86–1.99)	1.10	(0.71–1.70)	0.663
	≥3 to < 3	5.3	1.34	(0.99–1.79)	1.21	(0.90–1.64)	0.211
	≥3 to ≥3	6.0	**1.52**	**(1.03–2.22)**	1.21	(0.81–1.80)	0.354
Overweight	normal to normal	5.0			1		
	normal to overweight	5.6	1.13	(0.67–1.91)	1.06	(0.62–1.81)	0.833
	overweight to normal	5.2	1.04	(0.60–1.82)	0.89	(0.50–1.56)	0.678
	**overweight to overweight**	**2.1**	**0.41**	**(0.23–0.75)**	**0.39**	**(0.21–0.70)**	**0.002**
Irritability	rare to rare	2.5	1		1		
	rare to not rare	4.7	**1.91**	**(1.07–3.42)**	1.62	(0.90–2.94)	0.111
	not rare to rare	3.0	1.20	(0.64–2.34)	1.19	(0.62–2.30)	0.597
	**not rare to not rare**	**5.6**	**2.33**	**(1.36–3.98)**	**1.80**	**(1.03–3.13)**	**0.039**
Unwilling to attend school, rare to rare		3.9	1		1	1	
	rare to not rare	5.3	1.37	(0.98–1.91)	1.08	(0.76–1.52)	0.683
	not rare to rare	3.8	0.96	(0.66–1.40)	0.88	(0.60–1.30)	0.509
	not rare to not rare	7.1	**1.86**	**(1.35–2.56)**	1.35	(0.96–1.90)	0.093
Interact with parents, not rare to not rare		4.4	1		1		
	not rare to rare	5.1	1.15	(0.85–1.56)	1.20	(0.88–1.64)	0.254
	rare to not rare	4.3	0.97	(0.63–1.51)	1.04	(0.67–1.62)	0.858
	rare to rare	5.0	1.14	(0.81–1.59)	1.23	(0.87–1.74)	0.247

In multivariate model, sex and frequency of fruits and vegetable at baseline (phase 3) were also adjusted

*OR* odds ratio, *CI* confidence interval

Variables with a significant relationship to constipation are shown in bold

## Discussion

Our prospective study showed that, among Japanese adolescents aged 12 to 13 years, the incidence rate of constipation, defined as bowel movements “less frequently than once every 2 days,” was 4.7% over the 3-year period. Female sex, overweight (inversely), and infrequent consumption of fruits at baseline were significantly associated with adolescent constipation incidence. Furthermore, starting to skip breakfast, becoming physically inactive, and being persistently irritated were also associated with constipation incidence. Considering the fact that constipation is a common and persistent disease [19], our findings are valuable to decrease the burden of constipation in individuals and society. Our study was the first prospective study to demonstrate the incidence of adolescent constipation.

In our study, 6.8% of girls and 2.7% of boys developed constipation during the 3-year period (female OR 2.62, Table 2), which seemed comparable to the results of many cross-sectional studies showing that female adults are more likely to be constipated than male adults, although sex differences in the prevalence of constipation among children have been inconsistent [1, 7, 20, 21]. Some mechanisms of female predominance in constipation have been demonstrated. Total gut transit time was reported to be longer in women than in men [22]. Female sex hormones, such as progesterone, are thought to decrease the rate of small bowel and colonic transit times [23, 24].

The association between obesity or overweight and childhood constipation is inconsistent [25]. Previous studies conducted in clinical settings showed that children with functional constipation had a higher prevalence of obesity than controls [26, 27]. Meanwhile, most previous population-based studies for healthy children reported no association [21, 28] or an inverse association between obesity and constipation [8, 29, 30]. In our study, children who were overweight were less likely to develop constipation. One plausible explanation of this discrepancy is that children in population study may be less obese and have healthier bowel motility, i.e., which is more sensitive to food or a fecal mass than children studied in clinical settings.

The present study’s results showed that infrequent consumption of fruits was associated with constipation incidence. Insufficient dietary fiber intake is considered a major risk factor for constipation [1, 7]. Fiber is known to exert a beneficial effect on constipation, because it provides a fecal bolus mass-incrementing effect, possesses water retention properties, increases colon bacteria and gas production, and accelerates colon transit time [31]. Our prospective study in community adolescents demonstrated for the first time that adolescents having infrequent fruit intake develop constipation. Meanwhile, frequency of vegetable consumption did not predict constipation incidence in this study, which could be due to the following: first, most adolescents might have consumed vegetables almost every day at follow-up survey; thus, this questionnaire did not produce variance in our analysis; and second, fruit intake, rather than vegetable intake, might correspond more to actual fiber volume.

Notably, we focused on the impact of changes in lifestyle on constipation incidence (Table 3). Adolescents who started to skip breakfast during the three years were more likely to develop constipation. Similarly in previous cross-sectional studies in Japan, an association between skipping breakfast and constipation was reported in female college students [32, 33]. There have been several biological studies that can account for our result. Clock genes that form biological rhythms are functional in the liver, within gastrointestinal epithelial cells and neurons of the enteric nervous system [34]. Therefore, disruptions in circadian rhythmicity may lead to adverse health consequences [35]. In addition, Rao et al. conducted a study in healthy adults using manometry to investigate the motor activity of the colon [36]. The pressure waves in the colon during the night were inactive, but waking induced a threefold increase in motility, and meals induced a two-fold increase, proving that the ideal time for defecation would be after having breakfast in the morning. The importance of breakfast is not recognized as a predisposing factor of childhood constipation in recent reviews [1, 7], our findings in this prospective study can recommend, not anecdotally, that adolescents should keep having breakfast every day to prevent constipation.

Adolescents who became physically inactive were also more likely to develop constipation. Physical inactivity has been associated with constipation in many studies [1, 7]. The plausible mechanism by which physical activity modulates bowel movement is prolonged colonic transit time [37]. De Schryver et al. showed from their randomized controlled trial of 43 adults that subjects with programmed physical activity for 12 weeks have decreased rectosigmoid and total colonic transit time [38]. There were no significant associations between constipation and sleep duration or duration of TV viewing in the multivariate model. These factors may be confounded by other variables in our analyses.

Psychological stress, such as anxiety, irritability, infrequent parental interaction, and stressful life events, are well-known factors associated with childhood constipation [7, 8, 39]. To date, a large-scale study investigating the association between childhood constipation and psychological stress has been limited [40, 41], and the most studies were cross-sectional or retrospective in nature [1, 7]. We demonstrated that adolescents who had been persistently irritated more significantly developed constipation during the three years. Both adolescents who were irritated from grade 4 to grade 7 with “rare to not rare” and those with unwillingness to attend school “rare to not rare” and “not rare to not rare” were more likely to develop constipation. Contrary to the results from our cross-sectional study demonstrating that grade 4 children with infrequent child-parent interaction had a significant association with constipation [8], the frequency of the interaction showed no difference in the incidence of constipation. This discrepancy may be attributed to the psychological growth of adolescents. It may not be so important for adolescents to have frequent child-parent interactions compared to school-aged children. To prevent adolescent constipation, quality parental interactions that can mitigate adolescents’ psychological stress, rather than quantity, is recommended.

### Limitations

First, we conducted a questionnaire-based survey. Although obtaining psychological information from adolescents was a strength of this survey, it is subject to recall bias. Second, we did not collect information on gastrointestinal diseases and medication use. However, these conditions were not common among adolescents in the community. Third, other factors, such as genetic predisposition, volitional stool retention, menstruation, and microbiota were not included [42]. Finally, adolescents in our study were included from one prefecture in Japan. Thus, the results may differ in the general population because lifestyle factors such as diet and exercise are influenced by cultural differences. Further international, large-scale studies including these factors should be conducted.

## Conclusion

Approximately 5% of adolescents developed constipation during the 3-year study period, and the incidence was especially high in female (6.8%). Infrequent fruit intake, unhealthy lifestyle habits such as skipping breakfast and physical inactivity, and persistent psychological stress, were associated with the incidence of constipation. Beyond anecdotal evidence, maintaining a healthy lifestyle should be recommended for adolescents to prevent constipation. Additionally, health providers and parents should encourage adolescents to maintain a healthy lifestyle and avoid persistent psychological stress to reduce their risk of developing constipation.

## Supplementary Information

**Additional file 1.** Questionnaire in Toyama Birth Cohort Study (Phase 3).

## Data Availability

The dataset analyzed during the current study are not publicly available due to ethical restrictions and specific legal framework in Japan. It is prohibited by the Act on the Protection of Personal Information (Act No. 57 of 30 May 2003, amended on 9 September 2015) to publicly deposit data containing personal information. However, data are available from the corresponding author on reasonable request.

## Abbreviations

BMI: Body mass index
OR: Odds ratio
CI: Confidence internal
SPSS: Statistical package of social sciences
