# Supplementary material for: Lifestyle, psychological stress, and incidence of adolescent constipation: results from the Toyama birth cohort study
Source: BMC Public Health. 2021 Jan 6;21:47. doi: 10.1186/s12889-020-10044-5 (PMC7789557; doi:10.1186/s12889-020-10044-5)
Supplement: Supplementary file 1 — Additional file 1. Questionnaire in Toyama Birth Cohort Study (Phase 3). [file 12889_2020_10044_MOESM1_ESM.docx]

Additional file 1: Questionnaire in Toyama Birth Cohort Study (Phase 3)

On basic characteristics

Name Sex

Birth day Age

Hight cm Weight kg (Refer to the newest result obtained at school)

On lifestyles

Q. Do you have breakfast?

Every day, sometimes, or hardly.

Q. How often do you exercise or paly outside?

Very often, often, or rare.

Q. How long do you watch TV on a week day?

<2, <3, or ≥3 hours

Q. How long do you usually sleep?

hours

To parents or guardians

Q. Except school lunch, how many times does your child consume fruits and vigetables per week?

Fruits consumption

Almost every day, 3-5 times, or 0-2 times per week.

Vegetable consumption

Almost every day, 3-5 times, or 0-2 times per week.

On psychological status

Q. How often do you feel irritated?

Often, sometimes, rarely, or none

Q. How often do you feel like you do not want to go to school?

Often, sometimes, rarely, or none

Q. How often do you usually interact with your parents?

Often, sometimes, rarely, or none

On bowel movements

Q. How often do you usually have bowel movement?

At least once daily, once every 2 days, or less frequently than once every 2 days.
